# Microbial fuel cell biosensor for the determination of biochemical oxygen demand of wastewater samples containing readily and slowly biodegradable organics

**DOI:** 10.1007/s10529-020-03050-5

**Published:** 2020-11-27

**Authors:** Gábor Márk Tardy, Bálint Lóránt, Miklós Gyalai-Korpos, Vince Bakos, David Simpson, Igor Goryanin

**Affiliations:** 1 Department of Applied Biotechnology and Food Science, Budapest University of Technology and Economics, Szt. Gellért tér 4., Budapest, 1111 Hungary; 2 Pannon Pro Innovations Ltd., P.O.B 41, Budapest, 1400 Hungary; 3 BES Europe Ltd, 39 Murányi u., Budapest, 1078 Hungary; 4 Okinawa Institute of Science and Technology, 1919-1 Tancha, Onna-Son, Kunigami-gun, Okinawa, 904-0495 Japan; 5 School of Informatics, University of Edinburgh, 10 Crichton str., Edinburgh, EH8 9AB UK; 6 Tianjin Institute of Industrial Biotechnology, 32 West 7th Avenue, Tianjin Airport Economic Area, Tianjin, 300308 China

**Keywords:** Biochemical oxygen demand, Biosensor, Microbial fuel cell, Wastewater characterization

## Abstract

**Objectives:**

Single-chamber air cathode microbial fuel cells (MFCs) were applied as biosensors for biochemical oxygen demand (BOD) measurement of real wastewaters with considerable suspended and/or slowly biodegradable organic content.

**Results:**

The measurement method consists of batch sample injection, continuous measurement of cell voltage and calculation of total charge (Q) gained during the biodegradation of organic content. Diverse samples were analyzed: acetate and peptone samples containing only soluble readily biodegradable substrates; corn starch and milk samples with suspended and colloidal organics; real domestic and brewery wastewaters. Linear regression fitted to the Q vs. BOD_5_ measurement points of the real wastewaters provided high (> 0.985) R^2^ values. Time requirement of the measurement varied from 1 to 4 days, depending on the composition of the sample.

**Conclusions:**

Relative error of BOD measured in the MFCs comparing with BOD_5_ was less than 10%, thus the method might be a good basis for the development of on-site automatic BOD sensors for real wastewater samples.

**Electronic supplementary material:**

The online version of this article (10.1007/s10529-020-03050-5) contains supplementary material, which is available to authorized users.

## Introduction

In microbial fuel cells (MFCs), the chemical energy of biodegradable organics can be converted directly to electricity through the metabolism of exoelectrogenic bacteria. The generated electricity can be measured and/or utilized (Rabaey and Verstraete 2005). A promising perspective for the application of this technology is to create MFC-based biosensors for the detection and measurement of biodegradable organics and/or toxic materials in water or wastewater (Yang et al. 2015; Lóránt et al. 2019).

One of the most important parameters for the characterization of polluted water/wastewater samples is biochemical oxygen demand (BOD). With the main aim of simplifying the standard respirometry-based BOD_5_ measurement and reducing its time requirement of 5 days, numerous researches have been carried out recently to develop MFC-based BOD measurement methods (Spurr et al. 2018; Do et al. 2020).

In MFC biosensors, prompt assessment of BOD can be attempted based on the Monod-type relationship between the generated current and the actual biodegradable organic content in the anolyte (Lóránt et al. 2019). The result of this measurement method, however, refers only to the soluble readily biodegradable content, as the slowly biodegradable suspended organic fraction of real wastewaters needs considerable time (several hours to few days) to be hydrolyzed and biodegraded (Henze et al. 2015; Do et al. 2020; How et al. 2020). To take this fraction into consideration, total charge (Q) gained by the elimination of substrates has to be calculated within a longer time-frame (typically few hours to few days) of the biodegradation. Q was found to be in linear relationship with the BOD_5_ concentration (Liu et al. 2018; Wang et al. 2018). Although Q derived BOD may involve the measurement of suspended slowly biodegradable substrate content, most of the related references investigated basically soluble, readily biodegradable substrates (e.g. Wang et al. 2018; Gao et al. 2020). To our knowledge, MFC-based measurement of real wastewater samples with highly variable suspended organic content has not yet been evaluated in details, although it is a crucial point in the practical application of MFC BOD biosensors.

In the current research, single-chamber air cathode MFCs were applied as BOD sensors. The main aims of the research were: (1) to determine the relationship between the total charge and BOD_5_ for artificial wastewater samples with variable suspended slowly biodegradable organic content; (2) to verify the accuracy and reliability of the developed method by the investigation of real wastewater samples.

## Materials and methods

### MFC architecture and inoculation

**Fig. 1:**
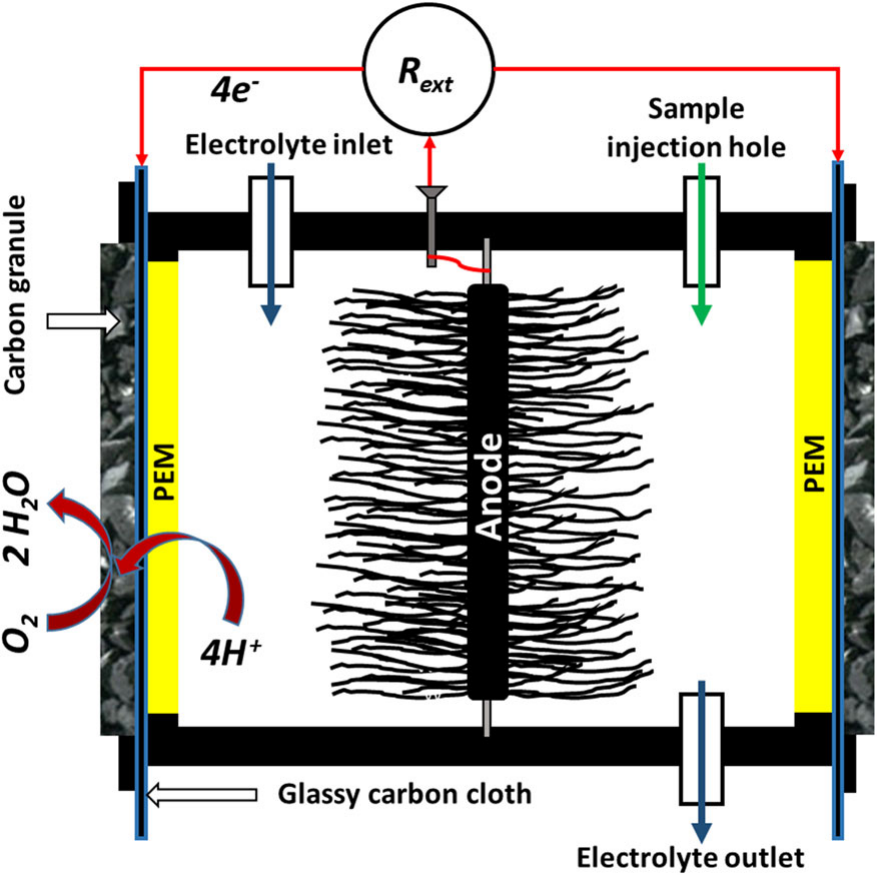
Scheme of the applied single-chamber air cathode microbial fuel cells (*R*_*ext*_ external resistance, *PEM* proton exchange membrane)

Three identical cubic-shaped single-chamber air cathode MFCs were applied as biosensors, with an inner volume of 230 cm^3^ (see Fig. 1). 10 cm diameter graphite brushes (made of Zoltek 3505015T-13 fiber) were used as anode. Porous ceramic plates with ion exchange polymer in the pores were used as proton exchange membranes (PEM). The air cathodes contained 25 ml of biomass-originated carbon granules, connected to the PEM through a conducting glassy carbon cloth layer with an interface of 48 cm^2^. More detailed description of the cells including basic performance characteristics can be found in our previous publication (Lóránt et al. 2019).

The electrolyte inlet and outlet were positioned on the opposite side of the anode chamber to facilitate its rinsing with a standard (substrate free) electrolyte. The flow was provided by a Masterflex® peristaltic pump. The composition of the standard electrolyte was as follows: 3.13 g NaHCO_3_ l^−1^, 0.31 g NH_4_Cl l^−1^, 0.13 g KCl l^−1^, 4.22 g NaH_2_PO_4_ l^−1^, 6.93 g Na_2_HPO_4_*12 H_2_O l^−1^ (based on Oh et al. 2004). A sealable sample injection hole was placed on the top of the anode chamber. The external circuit included the adjustable resistance and a data collection device (Graphtec midi logger GL840 oscilloscope).

The MFCs were inoculated with diluted primary settled sludge from domestic wastewater treatment plant (South-pest WWTP), with sodium acetate substrate in 40 mM concentration. Within a 2 weeks long inoculation period applying 1000 Ω external resistance, the voltage increased and stabilized at 0.5–0.6 V in each cell. Having the voltage reached its maximum plateau, the inoculating suspension was washed out with standard electrolyte, and 1 month of startup period was carried out using acetate substrate (40 mM) to reach stable operation prior to measurements.

### Basic sample quality measurements

BOD_5_ measurements and chemical oxygen demand (COD) measurements were carried out according to international standards (APHA 1999). In case of samples with considerable suspended organics content, dissolved COD concentrations (COD_D_) were also determined besides total COD. Preparation of samples for COD_D_ measurement was carried out by flocculation using ZnSO_4_ and consequent filtration as described by Mamais et al. (1993).

### Applied substrate materials

In order to investigate the reliability, evaluate and adapt the measurement with MFC biosensors to real wastewaters, series of samples containing substrates with different complexity and solubility were investigated (see in Table 1).

**Table 1:** Origin and description of the materials investigated in the MFCs

Name	Media type	Substrate content of the media	Concentration (mg BOD_5_ l^−1^)
Sodium-acetate (Molar chemicals)	Chemically defined	Soluble readily biodegradable	2600 (stock solution)
Peptone (Molar chemicals)	Complex	Soluble readily biodegradable	37,000 (stock solution)
Corn starch (Hungrana Ltd.)	Polysaccharide	Partly suspended partly soluble	12,000 (stock solution)
Milk (Alföldi Tej Ltd.)	Complex	Partly soluble, partly colloidal	80,000–110,000
Pre-clarified domestic wastewater (South-pest WWTP)	Complex	Soluble readily biodegradable and suspended slowly biodegradable	80–320
Brewery wastewater (Mad Scientist Craft Brewery)	Complex	Soluble readily biodegradable and suspended slowly biodegradable	59–660

Sample preparation of investigated materials were as follows:

- Acetate and Peptone (soluble substrates): stock solution was prepared and diluted to the desired concentrations by water before injection.
- Corn starch (partly suspended polysaccharide): stock suspension was prepared by suspending and stirring corn starch in water while heating it up to ~ 80 °C for 10 min to hydrate and partly dissolve the polysaccharide content. The stock suspension was diluted to the desired concentration by water prior to injection. COD and COD_D_ measurements showed, that in average ~ 20% of the sample’s total COD content was in dissolved form, while the rest was in suspended form.
- Milk (soluble and colloidal organic content): commercial milk was diluted with water before injection.
- Domestic and brewery wastewaters (soluble, colloidal and suspended organic content): Before the injection, some of the original wastewater samples were diluted by water in order to cover a wide BOD_5_ concentration range (59–660 mg BOD l^−1^), enabling the calculation of an appropriate Q vs. BOD_5_ correlation. Detailed analytics of real wastewaters are summarized in Supplementary Table 1.

### Procedure of the BOD measurement using MFC biosensors

Before each measurement, anode chambers of the cells were rinsed with 0.7 l of standard electrolyte to ensure the biodegradable substrate free state in the cell. Once the rinsing finished, the electrolyte flow was stopped and sample injection procedure started: before the introduction of the sample, equal volume of anolyte was vented from the MFCs, then the anode chamber was filled up with the sample by automatic pipette, and the measurement started. During method development it was revealed that the favorable amount of biodegradable organics introduced to the anodic chamber should be below 40 mg in BOD_5_ to result in an acceptable measurement time (< 4 d) and above 4 mg to enable an accurate measurement. Thus, the amount of the introduced substrate was adjusted: the volume of the injection was 4 ml in case of artificial samples and 60 ml in case of real wastewaters with lower initial BOD_5_ concentrations (see Table 1).

During experiments with acetate, external resistance (R_ext_) was set to 400 Ω as polarization curves suggested the maximum power output at that value. All other cases R_ext_ was set to 100 Ω to enhance the rate of biodegradation. During measurement, voltage on the external resistor was registered in every 5 min by the data collection device. At the beginning, voltage increased rapidly and generally reached a plateau. After some time (depending on the amount and quality of the sample), the voltage began to decrease as a result of substrate depletion. The end-point of the measurement was defined as a discrete voltage value determined during the development of the method (0.02 V in case of real wastewater samples and 0.05 V in case of all other samples). At these cutoff points the substrates were considered to be practically depleted.

Knowing the external resistance, current was calculated by Ohm’s law, and the current vs. time in the measurement time frame was numerically integrated as described by Eqs. (1, 2). The total charge obtained from biodegradation was calculated and compared with the BOD_5_ value of the sample:

1 $$I = \frac{U}{{R_{ext} }}$$

2 $$Q = \int_{{t_{s} }}^{{t_{e} }} {I\,dt}$$

where I is the current in the external circuit (A), U is the voltage (V) measured on the external resistance (R_ext_, Ω), Q is the total charge (C) flown through the external circuit during the measurement, t_s_ (s) is the starting time of the experiment, t_e_ (s) is the end time of the measurement. During the whole research, three identical MFCs were operated in parallel, referred in the paper as MFC “A”, “B” and “C”. Coulombic efficiencies were calculated from Q and the known COD values of the samples, based on Logan (2008).

## Results and discussion

### Verification of the measurement procedure with acetate substrate

**Fig. 2:**
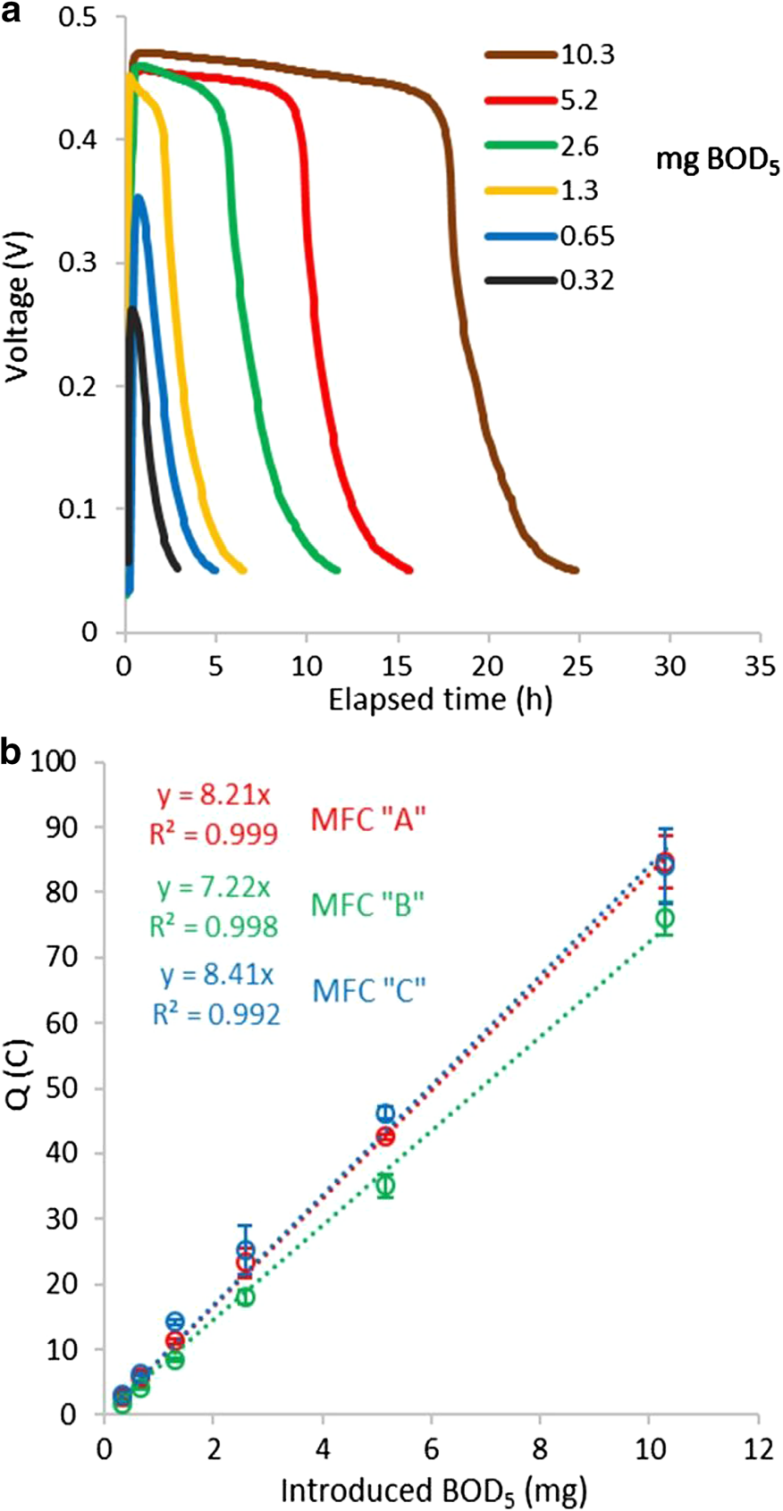
**a** Voltage vs. time curves with different amounts of introduced acetate (given in mg BOD_5_) in MFC “A”; **b** linear correlation of the total charge (Q) values vs. introduced acetate in mg BOD_5_ for each MFC (error bars = standard deviation of Q, n = 3–5)

Figure 2a shows the voltage vs. time curves with different amounts of acetate, proving that the measurement reproduced the results of similar studies (Modin and Wilén 2012; Wang et al. 2018) in the applied cells. ~ 0.01 V of substrate-free baseline of the voltage (related to endogenous respiration) was measured by maintaining 3 days long substrate-free state in the anode chambers. To shorten the measurement time, however, the end-point was considered to be 0.05 V. This consideration was based on the concentration dependence of the voltage in the cells (Lóránt et al. 2019), which suggested that at 0.05 V potential on the 400 Ω R_ext_, the remaining readily biodegradable substrate concentration in the anode chamber is as low as ~ 0.08 mg BOD_5_ l^−1^. As a result, the measurement time was shortened by ~ 12–24 h, still practically representing the complete biodegradation. Figure 2b shows that the calculated total charge values give good linear correlation with the BOD_5_ values of the samples: coefficient of determination (R^2^) is higher than 0.99 in each MFC. Small standard deviation and relative standard deviation (SD = 0.64; RSD = 8.0%) were found with the calibration line slopes of different MFCs. Coulombic efficiencies (CE) in the cells A, B and C were found to be 57%, 46% and 62% in average, with RSD below 10% in each cell, similar to the results of Wang et al. (2018).

With the applied 400 Ω R_ext_, the average substrate elimination rate was ~ 0.23 mg BOD_5_ l^−1^ h^−1^ with 5.2 mg introduced BOD_5_. In order to accelerate the biodegradation of the substrates, R_ext_ was reduced to 100 Ω. Due to this adjustment, the substrate elimination rate was increased considerably by 35%, from 0.23 to 0.31 mg BOD_5_ l^−1^ h^−1^ with the same amount of BOD_5_ spiked. At the same time, maximum voltage plateau remained above 0.25 V, still enabling reliable measurement. Thus, 100 Ω R_ext_ was used in further experiments.

### Evaluation of measurements with complex artificial media samples

**Fig. 3:**
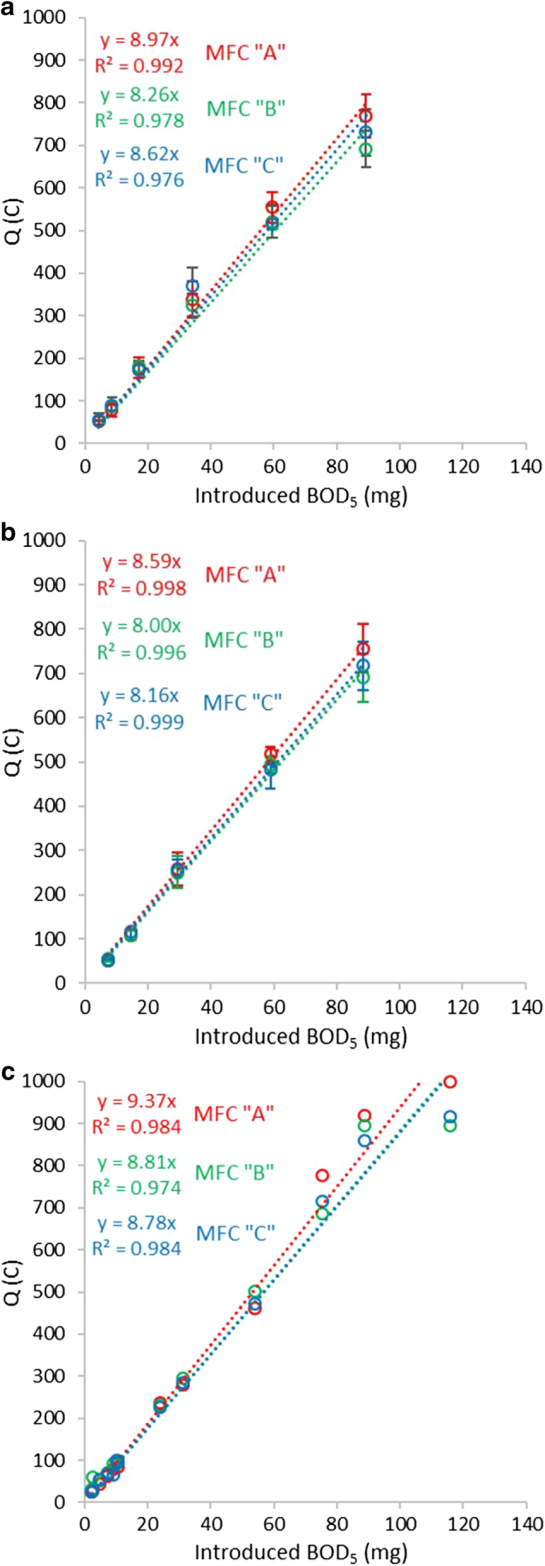
Linear correlation of the total charge values vs. introduced mg BOD_5_ in case of **a** peptone; **b** corn starch; **c** milk, for each MFC (error bars = standard deviation, n = 3–5)

On Fig. 3 Q vs. injected BOD_5_ correlations of three different complex artificial substrates (peptone solution, starch suspension, diluted milk) are depicted for the three MFCs. With the investigated substrates, linear correlations provided very high (> 0.97) R^2^ values in each cell. The slopes of calibration lines of the three MFCs proved to be similar with every substrate. Slope averages together with SD and RSD data, as well as the coulombic efficiency averages with similar statistical evaluation are summarized in Table 2. The low SD (≤ 0.4) and RSD (< 5%) values suggest that practically the same Q vs. introduced BOD_5_ calibration can be applied for MFCs with identical design and inoculation.

**Table 2:** Slope averages (C mg^−1^ BOD_5_) of the linear correlations of total charge (Q) vs introduced BOD_5_, and coulombic efficiency (CE) averages obtained for the three cells with standard deviation and relative standard deviation values

Substrate	Slope	SD	RSD (%)	CE (%)	SD	RSD (%)
Peptone	8.62	0.36	4.1	75.8	9.59	12.6
Corn starch	8.25	0.31	3.7	48.3	4.96	10.3
Milk	9.02	0.40	4.4	64.2	6.00	9.3

For the readily biodegradable soluble substrate of peptone, high (75.8%) coulombic efficiency was observed. The COD derived coulombic efficiency obtained for the corn starch samples containing large amount of suspended organics was considerably lower (48.3%), indicating that a larger part of the suspended content was not biodegraded in the measurement by exoelectrogenic metabolism. The slope of the Q vs. injected BOD_5_ correlation, however, is similar to those obtained with peptone (8.62 and 8.25, respectively, see Table 2). This suggests that similar fraction of starch suspension was biodegraded in the standard BOD measurement as in the MFC, which makes the two measurement comparable. Samples prepared with milk provided higher (64.2%) CE, with similarly low SD and RSD values. At the same time, the calculated average slope values of the linear regressions were very close to each other (8.62, 8.25, 9.02 C mg^−1^ BOD_5_ for peptone, corn starch and milk, respectively), with RSD < 5%. The time requirement of the measurements was 0.5–4 days depending on the injected amount of substrate. Based on these results it can be suggested that the BOD_5_ value of samples containing suspended biodegradable organics (~ 80% of the corn starch sample’s organic content) and/or colloidal complex substrates (e.g. the fat content of milk) can be reliably measured with the described method in the MFC biosensors.

### Evaluation of measurements with real wastewater samples

11 pre-clarified domestic wastewater samples and 10 brewery wastewater samples were investigated in the three MFCs (see Supplementary Table 1). As the biodegradable organic concentration of real wastewater samples were orders of magnitude lower than the applied stock solutions of the previously investigated artificial samples (see in Table 1), the injection volume was set to 60 ml (instead of 4 ml), to reach the applicable range of injected BOD_5_ from 4 to 40 mg.

The composition of the domestic and the brewery wastewater (Supplementary Table 1) was considerably different: average COD_D_/COD ratio of the domestic wastewater was 0.32, while that of the brewery wastewater was 0.63. Thus, larger part of the domestic wastewater’s organic content was in suspended form (presumably slowly biodegradable, based on Choi et al. (2005)), while the organic content of the brewery wastewater was predominantly in soluble (presumably readily biodegradable) form.

**Fig. 4:**
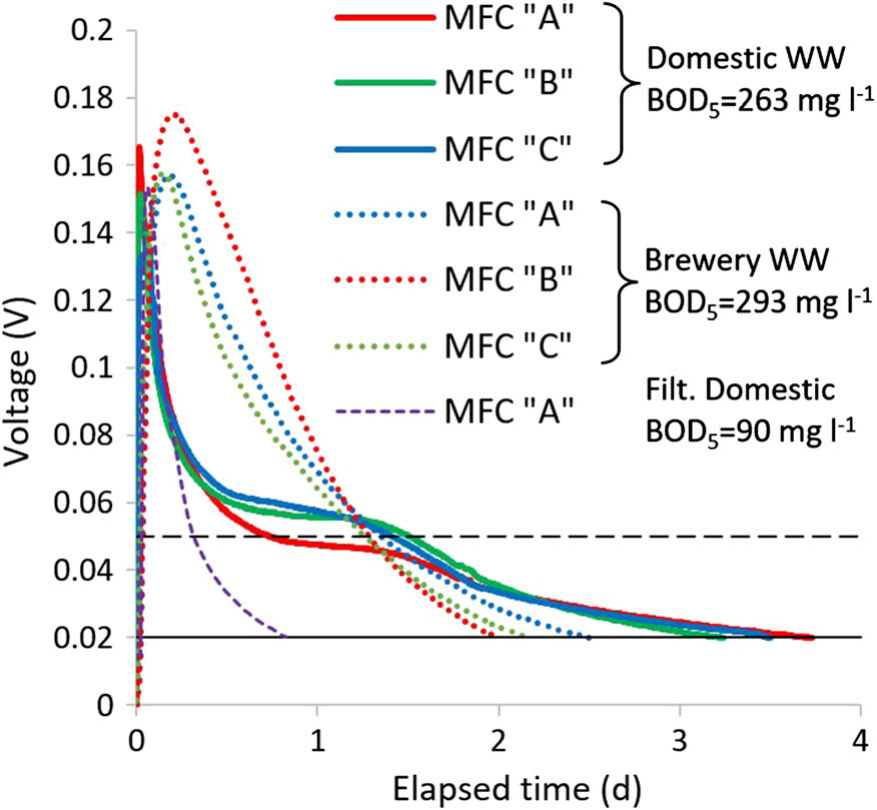
Typical shape of the voltage vs. time curve for domestic and brewery wastewaters obtained from the measurement of samples D3 and B6 (Supplementary Table 1). Dashed black line: 0.05 V cutoff potential previously applied for artificial samples; Solid black line 0.02 V cutoff potential applied for real wastewater samples. Purple dashed line stands for the result obtained in MFC “A” for the sample D3 filtrated before the measurement

The typical shape of the voltage vs. time curve was different with the two wastewaters, as it is depicted on Fig. 4, comparing two samples with similar amount of injected BOD_5_. The “two step” shape of the curves obtained with domestic wastewater has to be highlighted: it can be suggested that the first peak belongs to the biodegradation of soluble readily biodegradable substrates, while the following low-potential “shoulder” belongs to the hydrolysis and the consequent biodegradation of the suspended, slowly biodegradable compounds. This suggestion can be verified by comparing the curves of the original domestic wastewater samples with the same sample filtered before injection (not containing the suspended organics). The “two step” shape of the voltage vs. time curve is in accordance with the Activated Sludge Models (Henze et al. 2015) describing the biodegradation of the organic fractions of wastewaters. Most of the previous researches focused on samples without considerable suspended organic content (Modin and Wilén 2012; Commault et al. 2016; Wang et al. 2018; Gao et al. 2020), and to our knowledge, this “two step” characteristics of the voltage curve has not yet been described in the literature. As brewery wastewater contained biodegradable organics mainly in dissolved form, voltage peak supposedly related to the elimination of readily biodegradable organics was considerably wider than in case of domestic wastewater, and “two step” characteristics of the curve was not observed (see Fig. 4).

As the average potential of the “shoulder” obtained in the measurement of domestic wastewater samples was ~ 0.05 V in general, the previously determined 0.05 V cutoff boundary had to be reduced to ensure the measurement of the slowly biodegradable fraction. Therefore, the cutoff point with real wastewaters was considered to be the time when the potential fell to 0.02 V (two times higher than the 0.01 V baseline). In a previous research (Gao et al. 2020) the cutoff point of a similar measurement in dual chamber MFCs was determined by the maximum voltage drop. This method, however, proved to be appropriate only for the investigation of soluble substrates. The fixed 0.02 V cutoff potential resolves the problem of measurements with real wastewater samples.

**Fig. 5:**
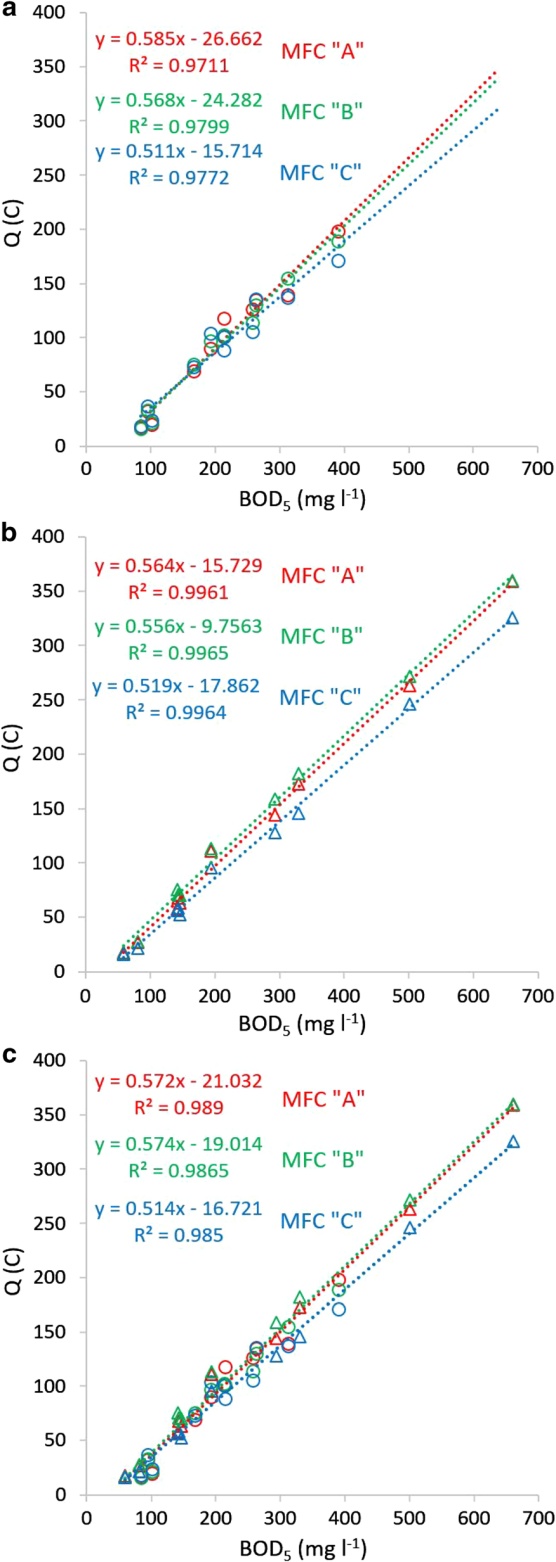
Linear correlation of the total charge values vs. BOD_5_ concentrations (mg l^−1^) of real wastewater samples obtained with the developed measurement, applying the 0.02 V cutoff voltage **a** domestic wastewater; **b** brewery wastewater; **c** combined correlation for brewery and domestic wastewaters (circles: domestic ww; triangles: brewery ww)

The Q vs. BOD_5_ correlation obtained for domestic and brewery wastewater samples proved to be linear with high (≥ 0.97) R^2^ values (see Fig. 5a, b). As the relative differences were lower than 4% between the slopes of the correlation lines obtained for domestic and brewery wastewaters and the average value (Supplementary Table 2), it can be suggested to use one unified calibration for the two different wastewaters (see Fig. 5c). The R^2^ values of these unified linear correlations are higher than 0.985. It means that by applying the described method, two wastewater types with considerably different composition can be appropriately measured with the same calibration line. The intersections of the unified linear regressions were 18.9 mg BOD_5_ l^−1^ in average, with 11% RSD. This derives from the fact, that with real wastewater samples below ~ 20 mg BOD_5_ l^−1^ concentration, the developed method does not produce voltage values higher than 0.02 V to calculate with, therefore this concentration can be considered as the limit of detection.

Depending on the concentration of the injected samples, the time requirement of the measurements varied from 0.5 to 4 days (see Supplementary Table 1). This is considerably longer than the 5–17.5 h obtained by Commault et al. (2016) in MFCs with 1 ml volume. Response time can be reduced by decreasing the size of the cell, however, it has its drawbacks: (1) due to possible inhomogeneity, the standard deviation of measurements originated from the sampling of liquid containing suspended solids can be very high when the sample volume is small, i.e. few milliliters (Rieger et al. 2010); (2) below 1 d measurement time the biodegradation of the suspended organics content is not ensured. MFC measured BOD (BOD_MFC_) concentrations were calculated from Q values by the obtained calibration. Comparing the BOD_MFC_ values with the BOD_5_ concentrations (measured by standard respirometric method) of the samples, the difference was less than 10% in average.

## Conclusion

Single-chamber air cathode microbial fuel cells were applied as biosensors for the determination of BOD of real wastewaters. Artificial sample series containing complex, suspended and colloidal organics (e.g. corn starch, milk) provided linear correlations with R^2^ > 0.97 for Q vs. injected BOD_5_, proving that besides soluble readily biodegradable substrates, the applied measurement method is appropriate for the determination of suspended and colloidal substrate content as well.

With the adapted method, two types of wastewaters (domestic, brewery) with significantly different composition can be measured using the same linear Q vs. BOD_5_ calibration (R^2^ > 0.985). The relative error of BOD values determined with the MFCs related to the BOD_5_ concentrations measured by standard respirometry method was less than 10%. As most single-chamber air cathode MFC designs require little maintenance and the sampling/rinsing steps can be easily automated based on the measured voltage signal, it can be suggested that this technology with the developed measurement method provides a promising candidate for on-site automatic BOD sensors.

Characteristics of the voltage vs. time curves obtained for domestic wastewater contains a higher voltage peak supposedly related to the biodegradation of readily biodegradable soluble substrates, and a lower voltage (~ 0.05 V) “shoulder” presumably belonging to the hydrolysis and consequent biodegradation of suspended slowly biodegradable organics. Thus, not only quantitive analysis of domestic wastewaters can be carried out with the developed method, but possibly the determination of soluble readily biodegradable and suspended slowly biodegradable substrate ratio as well. Further research is required in this matter.

## Electronic supplementary material

Below is the link to the electronic supplementary material.

Electronic supplementary material 1 (DOCX 23 kb)
