# Supplementary material for: Microbial fuel cell biosensor for the determination of biochemical oxygen demand of wastewater samples containing readily and slowly biodegradable organics
Source: Biotechnol Lett. 2020 Nov 27;43(2):445–54. doi: 10.1007/s10529-020-03050-5 (PMC7822768; doi:10.1007/s10529-020-03050-5)
Supplement: Supplementary file 1 — Electronic supplementary material 1 (DOCX 23 kb) [file 10529_2020_3050_MOESM1_ESM.docx]

**Supplementary Table 1** Composition of the domestic and brewery wastewater samples measured in the MFC sensors (COD_D_ = dissolved COD; BOD_in_ = amount of BOD_5_ (in mg) injected to the anode chamber of the cells)

| **Domestic wastewater** | |  |  |  |  |  |  |  |  |  |  |  |  |  |
| --- | --- | --- | --- | --- | --- | --- | --- | --- | --- | --- | --- | --- | --- | --- |
| Sample No. | 1 | 2 | 3 | 4 | 5 | 6 | 7 | 8 | 9 | 10 | 11 |  |  |  |
| COD (mg l^-1^) | 188 | 187 | 351 | 335 | 278 | 433 | 123 | 235 | 382 | 356 | 328 |  |  |  |
| COD_D_ (mg l^-1^) | 71 | 59 | 119 | 95 | 93 | 194 | 33 | 70 | 90 | 126 | 91 | Average | SD | RSD (%) |
| BOD_5_ (mg l^-1^) | 101 | 95 | 263 | 192 | 213 | 312 | 84 | 167 | 261 | 257 | 214 |  |  |  |
| BOD_in_ (mg) | 6.1 | 5.7 | 15.8 | 11.5 | 12.8 | 18.7 | 5.0 | 10.0 | 15.7 | 15.4 | 12.8 |  |  |  |
| COD_D_/COD | 0.38 | 0.32 | 0.34 | 0.28 | 0.34 | 0.45 | 0.27 | 0.30 | 0.24 | 0.35 | 0.28 | 0.32 | 0.06 | 18.5 |
| BOD_5_/COD | 0.54 | 0.51 | 0.75 | 0.57 | 0.77 | 0.72 | 0.68 | 0.71 | 0.68 | 0.72 | 0.65 | 0.66 | 0.09 | 13.1 |
| Average measurement time (d) | 0.9 | 1.1 | 3.5 | 3.3 | 2.8 | 3.7 | 2.7 | 3.0 | 3.9 | 3.8 | 2.9 |  |  |  |
|  |  |  |  |  |  |  |  |  |  |  |  |  |  |  |
| **Brewery wastewater** | |  |  |  |  |  |  |  |  |  |  |  |  |  |
| Sample No. | 1 | 2 | 3 | 4 | 5 | 6 | 7 | 8 | 9 | 10 |  |  |  |  |
| COD (mg l^-1^) | 250 | 497 | 188 | 834 | 197 | 439 | 124 | 87 | 736 | 304 |  |  |  |  |
| COD_D_ (mg l^-1^) | 159 | 263 | 98 | 537 | 115 | 300 | 76 | 51 | 580 | 221 |  | Average | SD | RSD (%) |
| BOD_5_ (mg l^-1^) | 146 | 330 | 143 | 660 | 142 | 293 | 81 | 59 | 501 | 194 |  |  |  |  |
| BOD_in_ (mg) | 8.8 | 19.8 | 8.6 | 39.6 | 8.5 | 17.6 | 4.9 | 3.5 | 30.1 | 11.6 |  |  |  |  |
| COD_D_/COD | 0.64 | 0.53 | 0.52 | 0.64 | 0.59 | 0.68 | 0.62 | 0.59 | 0.79 | 0.73 |  | 0.63 | 0.08 | 13.3 |
| BOD_5_/COD | 0.58 | 0.66 | 0.76 | 0.79 | 0.72 | 0.67 | 0.65 | 0.68 | 0.68 | 0.64 |  | 0.68 | 0.06 | 8.8 |
| Average measurement time (d) | 1.4 | 3.0 | 1.5 | 3.9 | 1.3 | 2.3 | 0.7 | 0.5 | 3.2 | 2.0 |  |  |  |  |

**Supplementary Table 2** Data and analysis of the linear regression parameters of the Q vs. BOD_5_ correlation for real wastewaters.

Slope = slope of the correlation line (C / mg BOD_5_ l^-1^)

Inters. = calculated intersection of the correlation line with the abscissa (mg BOD_5_ l^-1^)

Domestic = slope and intersection of the linear correlation fitted to domestic wastewater measurement points (see Fig. 5a)

Brewery = slope and intersection of the linear correlation fitted to brewery wastewater measurement points (see Fig. 5b)

Average = average value of slope and intersections obtained for domestic and brewery wastewaters

RD = relative difference between the slope of domestic and brewery wastewater related to the Average

D = difference between the intersection values obtained for domestic and brewery correlations

DOM+BREW = slope and intersection of the linear correlation fitted to all real wastewater measurement points (domestic and brewery together, see Fig. 5c)

|  | **Domestic** | | **Brewery** | | **Average** | | **RD (%)** | **D** | **DOM+BREW** | |
| --- | --- | --- | --- | --- | --- | --- | --- | --- | --- | --- |
|  | **Slope** | **Inters.** | **Slope** | **Inters.** | **Slope** | **Inters.** | **Slope** | **Inters.** | **Slope** | **Inters.** |
| **MFC "A"** | 0.585 | 45.584 | 0.564 | 27.873 | 0.575 | 36.729 | 3.6 | 17.710 | 0.572 | 21.032 |
| **MFC "B"** | 0.568 | 42.720 | 0.556 | 17.544 | 0.562 | 30.132 | 2.2 | 25.176 | 0.574 | 19.014 |
| **MFC "C"** | 0.511 | 30.733 | 0.519 | 34.416 | 0.515 | 32.575 | 1 | -3.683 | 0.514 | 16.721 |
| **Average** | 0.555 | 39.679 | 0.546 | 26.611 | 0.551 | 33.145 |  |  | 0.553 | 18.922 |
| **SD** | 0.039 | 7.878 | 0.024 | 8.507 | 0.031 | 3.335 |  |  | 0.034 | 2.157 |
